# Energy Balance of Triathletes during an Ultra-Endurance Event

**DOI:** 10.3390/nu7010209

**Published:** 2014-12-31

**Authors:** Anna Barrero, Pau Erola, Raúl Bescós

**Affiliations:** 1 National Institute of Physical Education, University of Barcelona, Barcelona 08038, Spain; 2 Departament d’Enginyeria Informàtica i Matemàtiques, Universitat Rovira i Virgili, Tarragona 43007, Spain; E-Mail: pau.erola@urv.cat; 3 Institute of Sport, Exercise and Active Living (ISEAL), College of Sport and Exercise Science, Victoria University, Melbourne 3011, Australia; E-Mail: raulbescos@gmail.com; 4 School of Health Professions, Faculty of Health and Human Sciences, University of Plymouth, Plymouth PL6 8BH, UK

**Keywords:** energy balance, triathlon, energy expenditure, energy intake, macronutrient consumption, endurance, body water

## Abstract

The nutritional strategy during an ultra-endurance triathlon (UET) is one of the main concerns of athletes competing in such events. The purpose of this study is to provide a proper characterization of the energy and fluid intake during real competition in male triathletes during a complete UET and to estimate the energy expenditure (EE) and the fluid balance through the race. Methods: Eleven triathletes performed a UET. All food and drinks ingested during the race were weighed and recorded in order to assess the energy intake (EI) during the race. The EE was estimated from heart rate (HR) recordings during the race, using the individual HR-oxygen uptake (*V*o_2_) regressions developed from three incremental tests on the 50-m swimming pool, cycle ergometer, and running treadmill. Additionally, body mass (BM), total body water (TBW) and intracellular (ICW) and extracellular water (ECW) were assessed before and after the race using a multifrequency bioimpedance device (BIA). Results: Mean competition time and HR was 755 ± 69 min and 137 ± 6 beats/min, respectively. Mean EI was 3643 ± 1219 kcal and the estimated EE was 11,009 ± 664 kcal. Consequently, athletes showed an energy deficit of 7365 ± 1286 kcal (66.9% ± 11.7%). BM decreased significantly after the race and significant losses of TBW were found. Such losses were more related to a reduction of extracellular fluids than intracellular fluids. Conclusions: Our results confirm the high energy demands of UET races, which are not compensated by nutrient and fluid intake, resulting in a large energy deficit.

## 1. Introduction

The popularity of ultra-endurance triathlon (UET) races (3.8-km swim, 180-km cycle, 42.2-km run) has greatly increased since the first Ironman was held in 1978 [1]. Given the long duration of these sport events, one of the main goals for athletes is to manage the consumption of food and drinks throughout the race [2] so as to enhance performance while maintaining body homeostasis. It has been estimated that the energy expenditure (EE) for a UET may range from 8500–11,500 Kcal [2,3,4]. However, to the best of our knowledge, only two previous studies have investigated this issue under field conditions. Kimber *et al.*, [3] assessed the energy balance of a UET using HR-oxygen uptake (*V*o_2_) regression equations during cycling and running as well as a multiple regression equation during the swimming section. They estimated an EE of 10,036 kcal and 8550 kcal in 10 males and 8 females, respectively. However, the energy intake (EI) was only 3940 kcal and 3115 kcal in both groups showing an energy deficit above 60% through the race. In a case study, Cuddy *et al.*, [5] combined two different approaches to assess the EE; indirect calorimetry and doubly labeled water. The data from both assessments were similar indicating that the EE of the athlete was ~9000 kcal. Nevertheless, this study did not assess the dietary consumption and fluid ingestion of the athlete during the event and, consequently, the EI and the energy deficit were not shown. Therefore, given this limitation of data, it seems important to address new research investigating the energy demands and the nutritional pattern of triathletes during real events.

Another key point for exercise performance in UET is fluid ingestion, which is not only important for performance, but also necessary to maintain a proper fluid homeostasis and to guarantee athlete’s health during ultra-endurance events [6]. Previous studies by Speedy *et al.* [7,8,9,10] have shown that triathletes performing a UET may suffer from exercise-associated hyponatremia even despite modest fluid intakes. This fact can be linked to renal function disturbances [11,12] which may induce an overload of extracellular water (ECW). However, changes in body mass (BM) during longer events are not only related with fluid balance. The reduction of the body stores of energy can also explain changes in the BM of athletes after ultra-endurance events. For instance, there is evidence indicating a significant decrease in muscle density (glycogen loss) and fat mass of athletes after a UET [13,14,15,16]. In addition, Laursen *et al.* [17] reported that a body mass loss of up to 3% was not linked with thermoregulatory failure in 10 triathletes performing a UET suggesting that part of the BM reduction occurred due to losses of glycogen and fat.

Accordingly, the main aim of this study was to provide a proper characterization of the energy and fluid intake of a group of male triathletes during a whole UET. A second aim was to estimate the EE and the fluid balance (intra and extracellular stores) of triathletes throughout the race using three different locomotion-specific individualized equations. We hypothesized that triathletes performing a UET would incur a substantial energy deficit of >70% due to the high energy demands of these types of events and the limited food intake of athletes through the race. We also hypothesized that sweat losses during UET under hot environmental conditions would not be compensated by fluid ingestion.

## 2. Experimental Section

### 2.1. Subjects

We placed an advertisement on the triathlon race webpage to recruit non-professional male triathletes. The inclusion criteria were to train at least 10 h per week and the participation in a minimum of one UET during the past 3 years.

Eleven triathletes volunteered to participate in the study (mean ± SD: age 36.8 ± 5.1 years, BM 75.5 ± 6.4 kg, height 1.74 ± 0.06 m, BMI 24.8 ± 1.7 kg/m^2^, maximal oxygen uptake (*V*o_2max_) 5.03 ± 0.4 L/min, 66.9 ± 4.1 mL/kg·min). Triathletes had an average of 10 ± 3 years of experience in UET and ultra-endurance events, and they had been training regularly for approximately 15–18 h per week for at least the three previous years. Before participating in the study, all subjects undertook a medical examination which included a physical examination (body composition), a medical questionnaire, and an electrocardiogram within the same year to ensure that each participant was in good health and gave their informed written consent, which was in accordance with legal requirements and the Declaration of Helsinki, and approved by the University of Barcelona’s Ethics Committee.

### 2.2. The Ultra-Endurance Triathlon

The triathlon was the *Extreme Man Salou-Costa Daurada triathlon*, an official race within the Catalan Triathlon Federation calendar composed of three stages consisting of a 3.8 km swim, 180 km cycle with a positive elevation over 2600 m and a 42.2 km marathon run.

The average (range) ambient temperature was 26 °C (13–30 °C), the water temperature was 21 °C (20.8–21.2 °C) and the relative humidity was 77% (64%–94%). The mean wind speed was 1.3 m/s (range 0.3–5 m/s). HR was monitored during the entire race using waterproof Polar RCX5 (Polar Electro, Kempele, Finland) portable monitors and averaged at 5 s intervals.

### 2.3. Preliminary Testing

Two weeks before the race, all subjects reported three times to the physiology laboratory (or to a swimming pool) to perform three incremental tests to volitional exhaustion in each discipline under randomised conditions separated by at least 48 h. The tests consisted of a graded swimming test in a 50-m pool, a graded cycling ergometry test and a graded treadmill running test [18]. Running and cycling tests were executed under controlled conditions (22 ± 1 °C, 40%–60% relative humidity, Pb 1013–1027 hPa). All the tests were performed at the same time of day to minimize the effects of circadian rhythms. Athletes were asked to refrain from caffeine, alcohol and heavy exercise on the day before the tests, and to report to the laboratory well hydrated after having eaten more than three hours before.

### 2.4. Nutritional Data

After the tests, triathletes were encouraged to follow their own diet scheduled usually in this sort of competition the days before and during the competition.

During the race, 25 trained researchers were divided among the refreshment points collecting all the wraps and bottles of each triathlete. Additionally, upon completion the UET, triathletes were asked to confirm the data collected during the event by researchers. Then, software was used to assess macronutrient intake (Centre d’Ensenyament Superior de Nutrició i Dietètica, University of Barcelona, Santa Coloma de Gramenet, Spain).

### 2.5. Body Mass and Bioimpedance Bioelectricity Variables

BM was measured using a Seca 710^®^ (Seca GmbH, Hamburg, Germany) weighing scale and TBW, ICW and ECW were measured using a multifrequency bioimpedance analyzer (Z-Metrix^®^, BioparHom^®^, La Motte Servolex, France) before and 30 min after the race (to avoid skin temperature effect on BIA).

### 2.6. Load of Exercise and Energy Expenditure

To estimate the total work load of exercise performed by each triathlete, we used the training impulse (TRIMP) method as previously described by Bescós *et al.* [19]. The individually derived linear relationship between HR and *V*o_2_ was used to estimate the oxygen cost during the work efforts for each segment. Three different individualized equations were established. These were three linear regression equations derived from data during each incremental exercise test. To estimate energy expenditure during the race, we used an energy equivalent of oxygen based on the mean intensity during racing time, as described in a previous study [20].

### 2.7. Statistical Analysis

Descriptive data is presented as mean ± standard deviation unless otherwise indicated. A one-way analysis of variance (ANOVA) was used to show differences between disciplines (swimming, cycling and running) during competition in the mean HR and percentage of time spent in each intensity zone as well as between macronutrient, fluid and sodium intake in each stage (cycling and running). Furthermore, another ANOVA test was performed to analyze differences between BIA data (TBW, ECW and ICW) before and after the race. Post-hoc analyses were performed using Tukey HSD. Pearson’s rank correlation analysis was used to assess the relationship between the individual physiological variables measured and performance in each stage of the triathlon, as well as the overall race time. A backward stepwise multiple linear regression analysis was used to determine the best predictors of final racing time after checking the correlation matrix for collinearity. Significance was set at *p* < 0.05 and all analyses were performed using PASW Statistics v 18 for Windows.

## 3. Results

### 3.1. Performance during the Ultra-Endurance Triathlon

All subjects successfully finished the race. Table 1 summarizes the main outcomes in each stage of the competition. As expected, time performed within zone I was significantly higher (69%) than in zone II (22%) and zone III (9%) (*p* < 0.001).

### 3.2. Macronutrient Intake

Food and fluids consumed during the UET were mainly those provided at the aid stations by the triathlon organizers. Table 2 summarizes the percent contribution from food and fluids consumed by athletes during the event.

Table 3 summarizes the consumption of macronutrients during the race. Subjects consumed 927 ± 178 g (6.2 ± 1.3 g/kg; 84 ± 18 g/h; 89.9% ± 3.5%) of carbohydrates (CHO), which provided the main source of energy consumed during the race (*p* < 0.001). The consumption of solid CHO (697 ± 147 g) was higher than the consumption of fluid CHO (229 ± 67 g; *p* < 0.001). Macronutrient intake was significantly greater in the cycling stage compared with the running stage (*p* < 0.001). However, regarding CHO, there were no statistical differences between both stages (cycling: 1.4 ± 0.5; running: 1.3 ± 0.3 g/min).

### 3.3. Fluid and Sodium Intake

Table 4 summarizes the fluid balance and the sodium intake during the UET. In absolute values, fluid and sodium intakes were significantly higher during the cycling stage than in the running stage (*p* < 0.001). However, when comparing relative values (fluid intake/time of exercise), fluid intake was significantly greater during the running period compared to the cycling stage ((395 ± 183) and (362 ± 172) mL/h, respectively; *p* < 0.001).

### 3.4. Energy Balance

**Figure 1:**
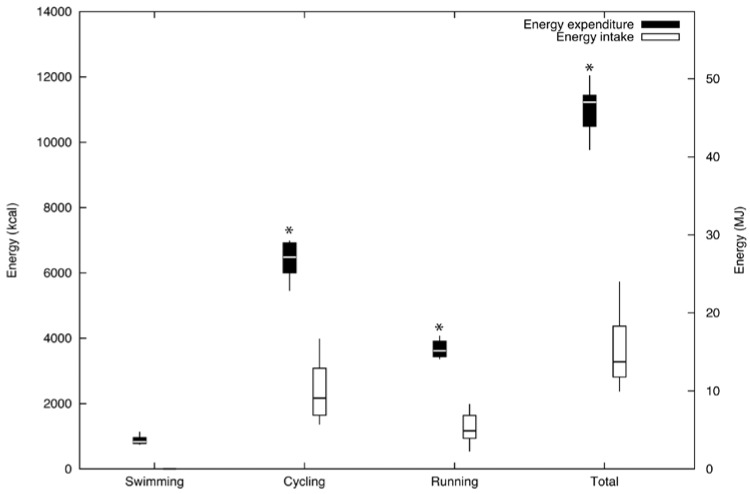
Swimming, cycling, running and overall energy intake and energy expenditure. Data are smallest value, first quartile (Q1), median, third quartile (Q3) and largest value of energy intake and energy expenditure during each stage and overall competition; * Energy expenditure was significantly greater than energy intake (*p* < 0.001).

Figure 1 shows the box-and-whisker plot of the estimated EI and EE during each stage of the competition and in overall terms. EE (11,009 ± 664 kcal; 46.1 ± 2.8 MJ) was significantly higher than EI (3643 ± 1219 kcal; 15.3 ± 5.1 MJ; *p* < 0.001) meaning that an energy deficit of 7365 ± 1286 kcal (30.8 ± 5.4 MJ; 66.9% ± 11.7%) occurred. Solid food significantly provided more energy than fluids (2812 ± 1150 kcal; 11.8 ± 4.8 MJ) (831 ± 668 kcal; 3.5 ± 2.8 MJ; *p* < 0.001). Absolute EI was higher during the cycling stage (2391 ± 82.9 kcal; 10.0 ± 0.4 MJ; 65.63%) compared to the running stage (1252 ± 43.1 kcal; 5.24 ± 0.18 MJ; 34.4%; *p* < 0.001). Mean ratios between EI and EE during the cycling, running and swimming stages (*i.e.*, including the swimming stage and transitions times) were 0.37 ± 0.14, 0.34 ± 0.13 and 0.33 ± 0.11, respectively.

**Table 1:** Swim, cycle, run and overall variables during the ultra-endurance triathlon race.

Stages	Racing Time (min)	TRIMP (a.u.)	Average HR (bpm)	Time Spent in Zone I (min)	Time Spent in Zone II (min)	Time Spent in Zone III (min)	Average Speed (km/h)
Swimming	63.1 (8.6)	186.0 (33.9)	149.0 (9.1)	4.0 (10.9)	0.01 (0.03)	60.7 (13.7)	3.7 (0.5)
Cycling	417.2 (38.4)	553.7 (109.7)	137.7 (5.4)	302.6 (84.2)	114.1 (76.8)	7.6 (20.5)	26.1 (2.3)
Running	257.3 (36.3)	313.9 (86.0)	133.9 (10.8)	209.2 (90.9)	51.7 (79.9)	0.4 (0.9)	10.0 (1.3)
Total	754.6 (68.8)	1053.6 (173.9)	136.7 (6.3)	515.8 (137.2) *	165.8 (138.0)	68.7 (22.4)	

Data are presented as mean (SD). TRIMP: training impulse (a.u., arbitrary units); HR: heart rate; *V*o_2_: oxygen uptake; Time spent in zone I: below to the first ventilatory threshold; zone II: between the first ventilatory threshold and the second ventilatory threshold; zone III: above to the second ventilatory threshold. * The time spent in zone I is significantly longer than the time value in zone II and zone III (*p* < 0.001).

**Table 2:** Percentage of energy contribution from food and fluids during the ultra-endurance triathlon race.

Food and Fluids	Energy Contribution (%)		
	Cycling	Running	Total
Sport gels	20.2	62.6	35.3
Sport bars	34.4	6.2	24.4
Sport drinks	20.3	13.5	17.9
Sandwich (parma jam and cheese)	13.9	2.0	9.7
Dried fruits (almonds and nuts)	4.2	3.6	4.0
Caffeinated drinks	1.9	6.0	3.3
Fruits (banana, apple and orange)	1.1	6.1	2.9
Cereals	3.1	-	2.0
Others (protein supplement)	0.8	-	0.5

**Table 3:** Macronutrient intake during the ultra-endurance triathlon race.

		Cycling	Running	Total
Carbohydrates	Solids (g)	405.7 (147.8)	291.5 (60.8)	697.2 (147.2) ^†^
	Fluids (g)	177.8 (67.0)	51.6 (24.3)	229.4 (67.0)
	Total (g)	583.5 (176.3) ^§^	349.0 (73.3)	926.6 (177.5) *
	g/kg ^a^	7.8 (2.3)	4.7 (1.2)	6.2 (1.3)
	g/h ^b^	84 (30)	78 (18)	84 (18)
	% ^c^	83.2 (5.6)	96.6 (3.8)	89.9 (3.5)
Proteins	Total (g)	40.7 (11.5) ^§^	4.6 (3.4)	45.4 (12.0)
	g/kg	0.6 (0.2)	0.1 (0.0)	0.3 (0.1)
	%	6.4 (2.9)	1.3 (1.0)	3.8 (1.6)
Lipids	Total (g)	69.1 (18.0) ^§^	7.7 (10.5)	76.8 (20.4)
	g/kg	0.9 (0.3)	0.1 (0.1)	0.5 (0.1)
	%	10.5 (3.6)	2.2 (3.0)	6.3 (2.4)

Data are presented as mean (SD). ^a^ Ratio between total macronutrient intake (g) and body mass (kg); ^b^ Ratio between total carbohydrate intake (g) and total racing time (min); ^c^ Macronutrient percentage of the total energy intake in each segment and in total; * The amount of carbohydrates was significantly higher than the amount of protein and lipids (*p* < 0.001); ^†^ The consumption of solid CHO was higher than that of fluid CHO (*p* < 0.001); ^§^ The intake amount was significantly higher in the cycling stage than in the running stage (*p* < 0.001).

**Table 4:** Fluid and sodium intake during the ultra-endurance triathlon.

		Cycling	Running	Total ^a^
Fluid intake (mL)		2530.9 (1255.9) *	1657.3 (717.4)	4188.2 (1836.9)
Fluid intake rate (mL/h)		361.6 (171.8) ^†^	394.5 (183.2)	366.6 (146.9)
Sodium (mg)	From fluids	485.2 (307.5) *	232.0 (208.5)	2152.2 (1124.2)
	From food	1135.9 (953.9) *	299.1 (369.3)	

Data are presented as mean (SD). ^a^ Includes time of swim and race transitions; * Values are significantly higher during cycling stage than in the running stage (*p* < 0.001); ^†^ Values are significantly lower during running stage than in the cycling stage (*p* < 0.001).

### 3.5. Body Mass and Bioimpedance Bioelectricity Variables

BM decreased significantly after the race (−4.3 ± 1.4 kg; −5.7% ± 1.9%, *p* < 0.001). Table 5 summarizes the differences in the TBW, ECW and ICW before competition and after competition. TBW, ECW and ICW also decreased significantly after the race (−8.4% ± 2.9%, −10.8% ± 3.7% and −6.8% ± 2.9%, respectively, *p* < 0.001). The decrease in ECW was significantly greater than the decrease in ICW (*p* < 0.001). The decrease in BM was closely related to the decrease in TBW (*r* = 0.98), ECW (*r* = 0.93) and ICW (*r* = 0.85) (*p* ≤ 0.001, respectively).

**Table 5:** Bioimpedance bioelectrical variables.

	Total Body Water		Extracellular Body Water		Intracellular Body Water	
	Liters	%	Liters	%	Liters	%
Pre-race	45.9 (3.4)	61.3 (3.8)	18.1 (1.3)	39.5 (0.7)	27.8 (2.2)	60.5 (0.7)
Post-race	42.0 (3.3) *	59.6 (4.0) *	16.1 (1.1) *	38.4 (1.2) *	25.9 (2.3) *	61.6 (1.2) *
Difference	3.8 (1.4)	8.4 (2.9)	2.0 (0.7)	10.8 (3.7)	1.9 (0.8)	6.8 (2.9)

Data are presented as mean (SD); * Lower values post-race compared to pre-race (*p* < 0.001).

### 3.6. Relationship between Racing Performance and Parameters Assessed during the Race

The decrease in TBW (*r* = 0.61, *p* = 0.046) and ECW (*r* = 0.72, *p* = 0.01) was related to the cycling time. CHO intake (g/min) was inversely related to the time (*r* = −0.71), and directly related to the speed (*r* = 0.70) during the cycling stage (*p* = 0.02). The stepwise multiple linear regression analysis identified the amount of CHO (g/min) ingested during the race and the % of ECW loss during the race (β = −0.52, SE = 60.8 and β = 0.47, SE = 4.4 respectively, *p* < 0.05) as the best predictors of total racing time, accounting for 60% (*r*^2^ = 0.60) of the final time variance.

## 4. Discussion

This study provided proper characterization of the energy and fluid intake, as well as the estimated energy expenditure, of a group of male triathletes during an entire ultra-endurance triathlon race. The estimated EE was ~11,000 kcal (46 MJ), whereas EI was only ~3600 kcal (15 MJ), which resulted in an energy deficit of almost 70% which partially confirms our first hypothesis.

### 4.1. Macronutrient Intake and Energy Balance

The high energy deficit can be explained by the lower EI of athletes in comparison with the higher energy demands of the current UET. In this study, athletes consumed an average of 927 ± 178 g of carbohydrates (90% of the overall EI). In relative terms, this amount corresponds to ~84 g/h. However, while these values can be considered within the current recommendations for longer events [21], it must be noted that CHO ingestion was heterogeneous between participants. For instance, five participants consumed less than 70 g/h of CHO while two of them ingested more than 90 g/h. The remaining four triathletes consumed an amount within the range of 70–90 g/h. Such differences between CHO ingestion were also linked with exercise performance on the bike. We found that triathletes ingesting higher amounts of CHO on the bike were able to perform better than those with low rates of CHO ingestion. This confirms the well-known fact that CHO supply plays a key role in improving exercise performance of athletes during endurance events [22].

Furthermore, another key point related to CHO intake is the type of CHO. It has been shown that the combination of different types of CHO (glucose-fructose-maltodextrin) with a consistent ratio (1:1:1) optimizes CHO oxidation and exercise performance [21]. Although it is difficult to estimate such ratio in this study due to the variety of food that athletes ingested during the event (Table 2), the combination of sport gels, sport bars, sport drinks and sandwiches may indicate that athletes ingested multiple CHO types.

The average protein intake by triathletes was 45 ± 12 g in total (4% of total EI). This amount was higher than previous data reported in triathletes [3]. However, to date, there is not a consensus about the protein needs during ultra-endurance events. Under laboratory conditions, it has been shown that the ingestion of 0.25 g/kg·h in combination with carbohydrates can improve protein balance by increasing protein synthesis and decreasing protein breakdown during long efforts (6 h) [23]. Considering the data provided by Koopman *et al.* [23], along with the amount of CHO intake by the athletes in this study, the “ideal” amount of protein ingestion would have been 236 g. This amount of protein would increase the energy intake (>900 kcal) and it would help to decrease the energy deficit of athletes.

The consumption of fat was 77 ± 20 g during the whole event. These values are lower compared with a previous study [3]. Like protein intake, currently there is no evidence supporting that the ingestion of lipids in form of either long-chain triacylglycerol (LCT) or tolerated amounts of medium-chain triacylglycerols increases performance during prolonged exercise [24]. Although the human body stores of fat are so large and will not become depleted after prolonged events, some stores such as intramyocellular triglycerides (IMTG) can play a key role during exercise [25]. These lipids are located directly at the site of contraction to ensure immediate substrate availability for physical activity [26]. Under conditions in which muscle glycogen availability is severely challenged, IMTG can compensate (at least in the endurance trained individual) by providing an alternative fuel source of similar potential energy to glycogen, to enable prolonged moderate intensity physical activity to be maintained [27]. However, while early research of the role of muscle glycogen in endurance exercise provided clear prescriptive information for the endurance-trained athlete, no such direction for optimizing exercise performance is yet apparent from research concerning IMTG. On the other hand, the inclusion of fat in the diet of ultra-endurance athletes could also be of interest in satisfying the taste of foods.

As shown by two previous studies [3,5], a high negative energy balance seems to be common in UET. The average of EE (11,009 ± 664 kcal) in this study was higher than in these previous studies. This fact could be explained by the positive elevation that triathletes had to overcome during the cycling stage in the current UET which induced a higher EE.

### 4.2. Body Mass, Fluid and Sodium Intake

In our athletes, the BM decreased significantly after the UET (−6% ± 2%). Decreases in BM up to as much as 11% of the total body weight have been shown previously [5,17,28,29,30]. These BM losses can be explained by high energy deficits which induce a decrease in muscle density (glycogen loss) and fat mass of the body of athletes participating in ultra-endurance events [13,14,15,16]. In the current study, we found a fluid deficit of 1.3 L which was far from the mean loss of body weight. Therefore, we assume that most of the loss in BM was due to losses of glycogen and fat stores.

Additionally, a significant and negative correlation between losses of TBW and ECW and performance during the cycling stage was found in this study. Although this fact corroborates the well-known fact that hydration is a key point for exercise performance, it is also important to note that losses of TBW were mainly linked to a reduction of ECW. An overload of ECW is a risk factor to develop hyponatremia [7,8,10]. Importantly, in the current study none of the athletes showed an increase of ECW. Furthermore, despite the fact that hyponatremia is an electrolyte disorder affecting serum sodium concentration (<135 mmol/L), the consumption of high amounts of sodium during exercise does not reduce the risk of developing hyponatremia [31]. Fluid overload is considered the main risk factor in the pathogenesis of hyponatremia and this is controlled by fluid intake during exercise as well as by the activity of renal hormones such as vasopressin [11,12]. The mean sodium intake reported by the current triathletes (2152 ± 1124 mg) was within the Recommended Dietary Intake and above the mean sodium ingestion indicated by Kimber *et al.* [3] in a previous study. This can be explained by the higher content of sodium of some sport food and sport drinks consumed by participants of this study during the event. None of the participants consumed sodium in the form of supplementation. This finding shows that it is possible to meet sodium recommendations through normal food and drinks in ultra-endurance events and, consequently, sodium supplementation is not needed. These data are in agreement with another recent study investigating sodium intake of ultra-endurance runners during a multi-stage race in a hot environment [32].

### 4.3. Strengths and Limitations

A major strength of this study is the careful nutritional analysis which was carried out in a community and setting where little information has been provided. Another strength is the testing during graded swimming that has not been done before with triathletes. However, we should also acknowledge some limitations and caveats in this study. Perhaps, the main limitation was the method used to estimate energy expenditure (relationship between HR-*V*o_2_). Despite, a good correlation between the doubly labelled water method (which is considered as the gold standard) and the use of equations based on the linear relationship between HR and *V*o_2_ under field conditions has previously been shown [5], it is also known that environmental factors such as dehydration and temperature can affect the linear relationship between HR and *V*o_2_ [33]. Studies investigating this issue in ultra-endurance athletes in laboratory conditions have shown that HR increases (during the first 6 h of exercise (cardiovascular drift)), but after that, this effect is compensated and HR progressively declines [34,35]. In regards to *V*o_2_ there are some discrepancies that need to be addressed in future research. While Mattson *et al.*, [35] found that *V*o_2_ consumption increased progressively (~16.6%) in the first 12 h of exercise and then remained stable during the next 12 h of exercise, Pokan *et al.* [34] showed a small increase (~4.3%) of *V*o_2_ over a similar test. In accordance with these data, it seems that we could have underestimated the EE of subjects of this study. Taking Mattson *et al.* [35] data, we have estimated that the athletes of our study could have spent between 700 and 800 more kcal in average during the event, and consequently, the EE could be even higher.

### 4.4. Practical Applications

In this study, we showed that triathletes were working mainly under the first ventilatory threshold where the body uses mainly fat as fuel and that the total EI did not provide the amount of energy necessary to deal with the UET, so the key seems to be looking for further adaptations to increase the ability to generate energy from fat. Accordingly, it should be useful to guide the training programs to increase the capacity to oxidize fat when muscle glycogen stores are depleted.

It would also be advisable to prepare the nutritional race strategy previously, according to the individual sweat rates and oxidation capacity.

## 5. Conclusions

The energy demands induced by UET exceed by far the energy intake of amateur triathletes despite consuming an adequate amount of CHO. The energy deficit induced by these events can be close to 70%. While an increase of lipid and protein consumption during UET would reduce the energy deficit of athletes, it is unknown how this would affect other key physiological responses such as gastric emptying and intestinal absorption during the race. Consequently, more research is needed in order to investigate the protein and lipid needs in ultra-endurance events. Furthermore, this study showed significant fluid losses (TBW, ECW and ICW) of athletes participating in the UET. A significant and negative relationship was found between losses of TBW and losses of ECW. Not only could the loss of body fluids potentially decrease exercise performance, it may also compromise the cardiovascular function and athlete’s health through the race.

## References

[1] Stiefel M., Rüst C., Rosemann T., Knechtle B. (2013). A comparison of participation and performance in age-group finishers competing in and qualifying for ironman hawaii. Int. J. Gen. Med..

[2] Laursen P.B., Rhodes E.C. (2001). Factors affecting performance in an ultraendurance triathlon. Sports Med..

[3] Kimber N., Ross J., Mason S., Speedy D. (2002). Energy balance during an ironman triathlon in male and female triathletes. Int. J. Sport Nutr. Exerc. Metab..

[4] Kreider R.B. (1991). Physiological considerations of ultraendurance performance. Int. J. Sport Nutr..

[5] Cuddy J., Slivka D., Hailes W., Dumke C., Ruby B. (2010). Metabolic profile of the ironman world championships: A case study. Int. J. Sports Physiol. Perform..

[6] O’Toole M.L., Douglas P.S. (1995). Applied physiology of triathlon. Sports Med..

[7] Speedy D.B., Noakes T.D., Kimber N.E., Rogers I.R., Thompson J.M., Boswell D.R., Ross J.J., Campbell R.G., Gallagher P.G., Kuttner J.A. (2001). Fluid balance during and after an ironman triathlon. Clin. J. Sport Med..

[8] Speedy D.B., Noakes T.D., Rogers I.R., Hellemans I., Kimber N.E., Boswell D.R., Campbell R., Kuttner J.A. (2000). A prospective study of exercise-associated hyponatremia in two ultradistance triathletes. Clin. J. Sport Med..

[9] Speedy D.B., Noakes T.D., Rogers I.R., Thompson J.M., Campbell R.G., Kuttner J.A., Boswell D.R., Wright S., Hamlin M. (1999). Hyponatremia in ultradistance triathletes. Med. Sci. Sports Exerc..

[10] Speedy D.B., Rogers I.R., Noakes T.D., Wright S., Thompson J.M.D., Campbell R., Hellemans I., Kimber N.E., Boswell D.R., Kuttner J.A. (2000). Exercise-induced hyponatremia in ultradistance triathletes is caused by inappropriate fluid retention. Clin. J. Sport Med..

[11] Rüst C.A., Knechtle B., Knechtle P., Rosemann T. (2012). No case of exercise-associated hyponatraemia in top male ultra-endurance cyclists: The “swiss cycling marathon”. Eur. J. Appl. Physiol..

[12] Shephard R.J. (2011). Suppression of information on the prevalence and prevention of exercise-associated hyponatraemia. Br. J. Sports Med..

[13] Knechtle B., Baumann B., Wirth A., Knechtle P., Rosemann T. (2010). Male ironman triathletes lose skeletal muscle mass. Asia Pac. J. Clin. Nutr..

[14] Knechtle B., Duff B., Amtmann G., Kohler G. (2008). An ultratriathlon leads to a decrease of body fat and skeletal muscle mass—The triple iron triathlon austria 2006. Res. Sports Med..

[15] Knechtle B., With A., Knechtle P., Rosemann T., Rüst C.A., Bescós R. (2011). A comparison of fat mass and skeletal muscle mass stimation in male ultra-endurance athletes using bioelectrical impedance analysis and different anthropometric methods. Nutr. Hosp..

[16] Mueller S.M., Anliker E., Knechtle P., Knechtle B., Toigo M. (2013). Changes in body composition in triathletes during an ironman race. Eur. J. Appl. Physiol..

[17] Laursen P.B., Suriano R., Quod M.J., Lee H., Abbiss C.R., Nosaka K., Martin D.T., Bishop D. (2006). Core temperature and hydration status during an ironman triathlon. Br. J. Sports Med..

[18] Barrero A., Chaverri D., Erola P., Iglesias X., Rodríguez F. (2014). Intensity profile during an ultra-endurance triathlon in relation to testing and performance. Int. J. Sports Med..

[19] Bescós R., Rodríguez F., Iglesias X., Knechtle B., Benítez A., Marina M., Padullés J., Vázquez J., Torrado P. (2011). Physiological demands of cyclists during an ultra-endurance relay race: A field study report. Chin. J. Physiol..

[20] Zuntz N. (1901). Ueber die bedeutung der verschiedenen nährstoffe als erzeuger der muskelkraft. Pflug. Arch. Eur. J. Physiol..

[21] Smith J.W., Pascoe D.D., Passe D.H., Ruby B.C., Stewart L.K., Baker L.B., Zachwieja J.J. (2013). Curvilinear dose-response relationship of carbohydrate (0–120 g·h^−1^) and performance. Med. Sci. Sports Exerc..

[22] Rodriguez N.R., di Marco N.M., Langley S. (2009). American college of sports medicine position stand. Nutrition and athletic performance. Med. Sci. Sports Exerc..

[23] Koopman R., Pannemans D.L.E., Jeukendrup A.E., Gijsen A.P., Senden J.M.G., Halliday D., Saris W.H.M., van Loon L.J.C., Wagenmakers A.J.M. (2004). Combined ingestion of protein and carbohydrate improves protein balance during ultra-endurance exercise. Am. J. Physiol. Endocrinol. Metab..

[24] Coyle E.F. (2004). Fluid and fuel intake during exercise. J. Sports Sci..

[25] Knechtle B., Müller G., Willmann F., Kotteck K., Eser P., Knecht H. (2004). Fat oxidation in men and women endurance athletes in running and cycling. Int. J. Sports Med..

[26] Van Loon L. (2004). Intramyocellular triacylglycerol as a substrate source during exercise. Proc. Nutr. Soc..

[27] Johnson N.A., Stannard S.R., Thompson M.W. (2004). Muscle triglyceride and glycogen in endurance exercise: Implications for performance. Sports Med..

[28] Hew-Butler T., Ayus J.C., Kipps C., Maughan R.J., Mettler S., Meeuwisse W.H., Page A.J., Reid S.A., Rehrer N.J., Roberts W.O. (2008). Statement of the second international exercise-associated hyponatremia consensus development conference, new zealand, 2007. Clin. J. Sport Med..

[29] Sharwood K.A., Collins M., Goedecke J.H., Wilson G., Noakes T.D. (2004). Weight changes, medical complications, and performance during an ironman triathlon. Br. J. Sports Med..

[30] Speedy D.B., Faris J.G., Hamlin M., Gallagher P.G., Campbell R.G.D. (1997). Hyponatremia and weight changes in an ultradistance triathlon. Clin. J. Sport Med..

[31] Hew-Butler T., Sharwood K., Collins M., Speedy D., Noakes T. (2006). Sodium supplementation is not required to maintain serum sodium concentrations during an ironman triathlon. Br. J. Sports Med..

[32] Costa R.J.S., Teixeira A., Rama L., Swancott A.J.M., Hardy L.D., Lee B., Camões-Costa V., Gill S., Waterman J.P., Freeth E.C. (2013). Water and sodium intake habits and status of ultra-endurance runners during a multi-stage ultra-marathon conducted in a hot ambient environment: An observational field based study. Nutr. J..

[33] Hiilloskorpi H., Fogelholm M., Laukkanen R., Pasanen M., Oja P., Manttari A., Natri A. (1999). Factors affecting the relation between heart rate and energy expenditure during exercise. Int. J. Sports Med..

[34] Pokan R., Ocenasek H., Hochgatterer R., Miehl M., Vonbank K., von Duvillard S.P., Franklin B., Wurth S., Volf I., Wonisch M. (2014). Myocardial dimensions and hemodynamics during 24-h ultraendurance ergometry. Med. Sci. Sports Exerc..

[35] Mattsson C.M., Enqvist J.K., Brink-Elfegoun T., Johansson P.H., Bakkman L., Ekblom B. (2010). Reversed drift in heart rate but increased oxygen uptake at fixed work rate during 24 h ultra-endurance exercise. Scand. J. Med. Sci. Sports.

